# Dietary ω-3 Fatty Acid Supplementation Improves Murine Sickle Cell Bone Disease and Reprograms Adipogenesis

**DOI:** 10.3390/antiox10050799

**Published:** 2021-05-18

**Authors:** Maria Teresa Valenti, Alessandro Mattè, Enrica Federti, Mark Puder, Lorenzo Anez-Bustillos, Michela Deiana, Samuele Cheri, Arianna Minoia, Carlo Brugnara, Maria Luisa Di Paolo, Luca Dalle Carbonare, Lucia De Franceschi

**Affiliations:** 1 Department of Medicine, University of Verona and Azienda Ospedaliera Universitaria Integrata Verona, 37128 Verona, Italy; mariateresa.valenti@univr.it (M.T.V.); alessandro.matte@univr.it (A.M.); enrica.federti@univr.it (E.F.); michela.deiana@univr.it (M.D.); samuele.cheri@univr.it (S.C.); arianna.minoia@univr.it (A.M.); lucia.defranceschi@univr.it (L.D.F.); 2 Department of Surgery and The Vascular Biology Program, Boston Children’s Hospital, Harvard Medical School, Boston, MA 02115, USA; mark.puder@childrens.harvard.edu (M.P.); lanezbus@bidmc.harvard.edu (L.A.-B.); 3 Departments of Pathology and Laboratory Medicine, Boston Children’s Hospital, Harvard Medical School, Boston, MA 02115, USA; carlo.brugnara@childrens.harvard.edu; 4 Department of Molecular Medicine, University of Padua, 35138 Padua, Italy; marialuisa.dipaolo@unipd.it

**Keywords:** sickle cell disease, bone histomorphometry, osteogenesis, adipogenesis, miRNAs

## Abstract

Sickle cell disease (SCD) is a genetic disorder of hemoglobin, leading to chronic hemolytic anemia and multiple organ damage. Among chronic organ complications, sickle cell bone disease (SBD) has a very high prevalence, resulting in long-term disability, chronic pain and fractures. Here, we evaluated the effects of ω-3 (fish oil-based, FD)-enriched diet vs. ω-6 (soybean oil-based, SD)- supplementation on murine SBD. We exposed SCD mice to recurrent hypoxia/reoxygenation (rec H/R), a consolidated model for SBD. In rec H/R SS mice, FD improves osteoblastogenesis/osteogenic activity by downregulating osteoclast activity via miR205 down-modulation and reduces both systemic and local inflammation. We also evaluated adipogenesis in both AA and SS mice fed with either SD or FD and exposed to rec H/R. FD reduced and reprogramed adipogenesis from white to brown adipocyte tissue (BAT) in bone compartments. This was supported by increased expression of uncoupling protein 1(UCP1), a BAT marker, and up-regulation of miR455, which promotes browning of white adipose tissue. Our findings provide new insights on the mechanism of action of ω-3 fatty acid supplementation on the pathogenesis of SBD and strengthen the rationale for ω-3 fatty acid dietary supplementation in SCD as a complementary therapeutic intervention.

## 1. Introduction

Sickle Cell Disease (SCD) is a genetic disorder due to the presence of a pathologic form of hemoglobin (Hb), Hb S, which originated in Africa and currently affects millions of patients worldwide. Although progress have been made in the clinical management of patients with SCD, the mortality and morbidity of young adults with SCD is still high when compared to a matched healthy population [1,2]. The severity of sickle cell related organ damage deeply affects the survival rate of patients with SCD [1,2,3]. Indeed, global burden disease studies have shown that SCD increases years lived with disability [3]. Among chronic sickle related complications, sickle cell bone disease (SBD) has a very high prevalence, leading to long-term disability, acute and chronic pain, and fractures [4,5]. Although progress have been made on mechanisms involved in sickle cell related organ damage, much still remains to be investigated on the pathogenesis of SBD [6,7,8]. In a humanized mouse model for SCD, we recently showed that SBD is generated by the imbalance between osteoclastogenesis and osteoblastogenesis, with a relative reduction in osteoblasts’ recruitment and activity [6]. This is associated with local up-regulation of pro-inflammatory cytokines such as IL-6 and antioxidant systems such as peroxiredoxin-2 [6].

In SCD mice, we recently showed defective pro-resolving events that might benefit from either ω-3 fatty acid supplementation or resolving D1 administration [9,10]. Studies in animal model of post-menopausal osteoporosis supplemented with ω-3 fatty acids have shown an improvement of bone homeostasis and a reduction in bone turn-over [11,12,13,14,15,16]. Two possible mechanisms have been invoked: reduction in osteoclast activity and/or increased osteoblast recruitment/activity [14,17]. It is worth noting that fatty acids have been suggested to be important in mesenchymal stem cell induction to adipogenesis, affecting bone health [18,19,20,21,22]. As osteoblast and adipocyte originate from a common precursor, a reduction in adipogenesis is required to ensure an adequate number of osteoblasts for bone formation. Since a reciprocal relationship between osteogenesis and adipogenesis occurs, increased adipogenesis is induced at the expense of osteoblastogenesis, resulting in reduce bone mass [23,24]. This helps with the understanding of the adipogenesis studies of ω-3 fatty acid supplementation, in which it ameliorates bone mineral density (BMD) and bone mineral content (BMC) [25]. The protective effects of ω-3 fatty acid supplementation in animal models of osteoporosis may be based on anti-inflammatory/pro-resolving and antioxidant effects, which are known to counteract age dependent bone loss and adipogenesis [25,26].

Although the mechanism of action of fatty acids on bone homeostasis is only partially known, growing evidence indicates the importance of GPR40, a free fatty acid receptor, which plays an important role in preventing bone loss by modulating osteoclast activity and increasing osteoblast activity throughout the osteoprotegerin (OPG) system [27,28,29].

With the intent to determine if ω-3 fatty acid supplementation might affect SBD, we present here studies on humanized SCD mice, treated with ω-6 (soybean oil-based, SD)- or ω-3 (fish oil-based, FD)-enriched diets and exposed to recurrent hypoxia/reoxygenation (rec H/R), an established modality to induce SBD in this mouse model. We provide several lines of evidence for a beneficial effect of ω-3 supplementation, which include prevention of the impairment of bone microarchitecture and bone loss induced by Rec H/R in SCD mice, increased osteoblasts recruitment/differentiation and reduced osteoclasts activity. We found that ω-3 fatty acid supplementation might re-program adipogenesis from white to brown/beige adipose tissue, which has a beneficial impact on bone health. Our results provide new insights on the molecular mechanism of action of ω-3 fatty acid supplementation on SBD and highlight the rationale for ω-3 fatty acid dietary supplementation as an additional therapeutic intervention for SCD.

## 2. Materials and Methods

### 2.1. Mice and Design of the Study

Experiments were performed on 4–6 week-old healthy control (*Hba^tm1(HBA)Tow^* *Hbb^tm3(HBG1,HBB)Tow^*) and SCD (*Hba^tm1(HBA)Tow^* Hbb^tm2(HBG1,HBB*)Tow^) mice [15,16]. The animal protocol was approved by Animal Care and Use Committee of the University of Verona (CIRSAL), “Ethical Approval Code” 56DC9.12. The details of the study design are reported in the Supplementary Methods section. To avoid possible confounding contributions of vitamin D deficiency on bone homeostasis, Vitamin D was present in mouse diet as standard diet supplementation of 1045 U/kg vitamin D_3_ [6]. Hematologic parameters and red cell indices were determined as previously reported [9,10,30]. Serum CTX and plasma MMP9 were measured as previously reported [6,31].

### 2.2. Measurements of Bone Homeostasis and Turnover

Bone histomorphometry and bone turnover was carried out as previously reported [6]. Details are reported in the Supplementary Methods section.

### 2.3. Bone Total RNA and microRNA Extraction and Reverse Transcription

Total RNA was extracted from femur as previously reported [6]. Homogenized bone was treated with the RNeasy Mini Kit (Qiagen, Hilden, Germany) and miRNeasy Mini Kit, according to the manufacturer’s instructions. RNA was quantified (260 nm) and checked for purity (260/280). First-strand complementary DNA (c-DNA) synthesis was performed using the Hight Capacity cDNA Reverse Transcription Kit (Applied Biosystems, CA, USA) and with TaqMan™ MicroRNA Reverse Transcription Kit (Applied Biosystems, CA, USA) according to the manufacturer’s protocol. Realtime PCR were performed as previously reported [6]. Details are reported in the Supplementary Methods section.

### 2.4. Bone Immunohistochemistry and Bone Immune-Microscopy

Tissue sections from Formalin-fixed paraffin-embedded (FFPE) bone slices were incubated overnight at 4 °C with Perilipin XP^®^ Rabbit mAb (D1D8) (Cell Signaling Technology, WZ Leiden), rinsed with PBS for 10 min and antibody detection was performed with the UltraVision ONE Detection System HRP Polymer and DAB Plus Chomogen (RTU) (Thermo Fisher Scientific, MA, USA). The samples were counterstained with Mayer’s Haematoxylin and observed under observed using a Leica DM2500 microscope (Leica Microsystems, Wetzlar, Germany). To evaluate the staining grade in a semiquantitative way, we applied the immunoreactive score (IRS) described by Remmele and Stegner [6]. The IRS is expressed as the product of staining intensity (between negative = 0 and strong = 3) and the percentage of positive stained cells (between 0 and 4; 0 = 0%, 1 = <10%, 2 = 10–50%; 3 = 51–80%, and 4 = >80%, respectively).

### 2.5. Colony Forming Units Osteogenic and Adipogenic Assays

The extract from femur biopsies was filtered using a 40 µm cell-strainer (Corning, NY, USA) and cells were cultured and differentiated as previously described [6]. Details are reported in the Supplementary Methods section.

### 2.6. Total RNA Extraction and Reverse Transcription of CFU-Osteoblasts or CFU-Adipocytes

Pellets from differentiated cells were collected and stored at −80 °C. Then, nucleic acid extraction was performed using the RNeasy Protect Mini Kit (Qiagen, Hilden, Germany), following the manufacturer’s protocol. RNA samples were quantified by Qubit 3 Fluorometer using Qubit RNA HS Assay Kit (Applied Biosystems, CA, USA). RNAs (2 µg) were reverse transcribed with the High-Capacity cDNA Reverse Transcription Kit (Thermofisher Scientific, Waltham, MA, USA), according to the manufacturer’s instructions.

### 2.7. Statistical Analysis

The 2-way ANOVA algorithm for repeated measures was used for data analysis. Differences with *p* < 0.05 were considered significant.

## 3. Results

### 3.1. FD Supplementation Improves Bone Structure and Reduces Bone Turnover in Humanized Sickle Cell Mice

**Figure 1:**
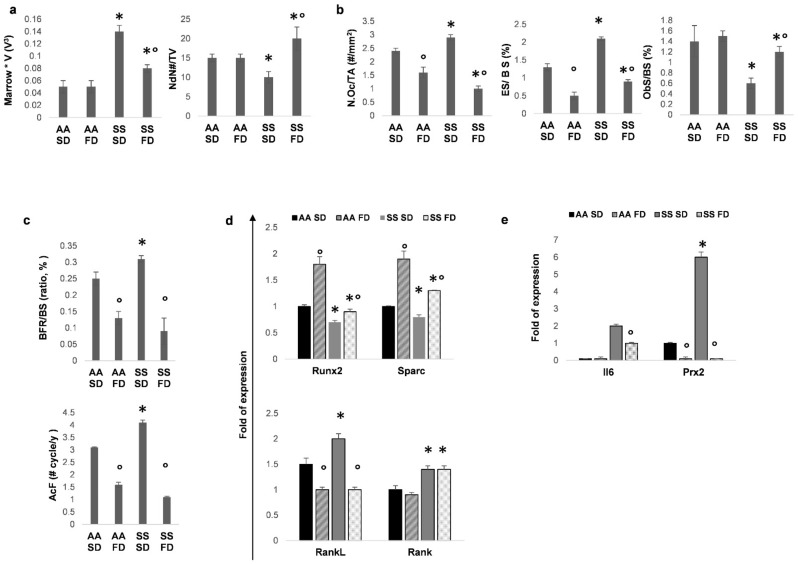
FD improves bone microarchitecture and reduces bone turnover in humanized sickle cell mice under normoxia. Quantitative histomorphometric analysis of distal femur of healthy (AA) and sickle cell (SS) mice under either SD or FD supplementation under normoxia. (**a**) Marrow Star Volume (Marrow*V) and increased Node Number (NdN#/TV) in healthy (AA) and sickle cell (SS) mice under either SD or FD supplementation. (**b**) Osteoclast number (N.Oc/TA), activity (ES/BS) osteoblastic activity (ObS/BS) in healthy (AA) and sickle cell (SS) mice under either SD or FD supplementation. (**c**) Bone Formation Rate (BFR/BS) and Activation Frequency (AcF), data are shown as mean ± standard deviation (SD). (**d**) Real-time PCR analysis of bone Runx2, Sparc, RankL, Rank expression in healthy (AA) and sickle cell (SS) mice under either SD or FD supplementation. (**e**) Real-time PCR analysis of interleukin- 6 (Il-6) and peroxiredoxin-2 (Prx2) bone expression in healthy (AA) and sickle cell (SS) mice under either SD or FD supplementation. (**a**–**e**) Data are shown as mean ± standard deviation (SD) (*n* = 4; 2 females and 2 males). * *p* < 0.05 compared to AA; ° *p* < 0.05 compared to SD.

In SS mice, FD significantly (i) reduced marrow star volume (MSV), (ii) increased Node Number (NdN#/TV) and (iii) decreased trabecular separation compared to SD- SS animals (Figure 1a, Figure S1).

The osteoclast number (N.Oc/TA) and activity, evaluated as the (ES/BS) ratio, were decreased in FD supplemented SS mice compared to the SD group (Figure 1b). Osteoblastic activity (ObS/BS) was higher in FD supplemented SS animals than in SD SS mice (Figure 1b). It is worth noting that we observed a significant reduction in the Bone Formation Rate (BFR/BS) and Activation Frequency (AcF) when compared to SD SS mice (Figure 1c). In healthy animals under either SD or FD supplementation, we did not find any changes in parameters of bone microarchitecture such as NdN#/TV (Figure 1a). We observed a significant reduction in osteoclast number and activity also in FD AA mice, whereas no change in osteoblast number or activity was observed in AA mice between SD and FD treated mice (Figure 1b). FD supplementation was associated with a lower bone turnover expressed as BFR/BS and AcF compared with the SD AA mouse group (Figure 1c). The differences in bone microarchitecture and bone turnover between AA and SS mice under SD are in agreement with our previous report on the same mouse strains using a standard diet with 1.3% total monosaturated fatty acids and 3.4% total polyunsaturated fatty acids (Figure 1a–c) [6].

We then explored the expression of genes linked to bone homeostasis. FD supplementation increased the osteogenic transcription factor Runx2 and its downstream gene Sparc, a marker of osteoblastic maturation. In both AA and SS mice, we found reduced expression of Rankl, an osteoclastic activating cytokine, when compared to SD groups (Figure 1d). The expression of the osteoclast receptor Rank was not affected by FD diet even if the levels were increased in SS compared to AA mice independently of diet supplementation, which is in agreement with our previous report (Figure 1d) [6]. The difference in expression of genes related to bone homeostasis between AA and SS mice under SD is in agreement with our previous report on the same mouse strains under standard diet (Figure 1a–c) [6]. Since pro-inflammatory and pro-oxidant local environment is an important determinant of osteoclast/osteoblast activity, we evaluated the expression of bone IL6, a bone pro-resorption cytokine cooperating with RankL, and of Prx2, an antioxidant enzyme involved either in osteoclastogenesis and osteoblastogenesis [6]. As shown in Figure 1e, the expression of IL6 was significantly reduced by FD supplementation in SS mice, while Prx2 expression was downregulated in both AA and SS FD mouse groups.

These data indicate that in SS mice under normoxia, FD improves osteoblast recruitment/differentiation and reduces osteoclast activity, contributing to decreased bone turnover compared to SS mice under SD.

### 3.2. In SCD Mice Exposed to Recurrent Hypoxia/Reoxygenation, FD Protects Bone Microarchitecture and Reduces Bone Turnover

Next, we investigated the effects of FD diet in mice exposed to recurrent H/R (Rec H/R) stress, mimicking the natural history of sickle cell related bone disease [6].

Complete blood count revealed no changes in either Hct or Hb, with only a significant reduction in HDW, a marker of dense red cells, in Rec H/R FD SS mice compared to Rec H/R SD SS animals (Figure S2a). Absolute Neutrophil counts were also lower in Rec H/R FD SS than in Rec H/R SD SS animals, supporting a systemic anti-inflammatory effect of FD in SS mice (Figure S2b). This agrees with our previous report in the same mouse model supplemented with either SD or FD and exposed to a single acute H/R session [10].

**Figure 2:**
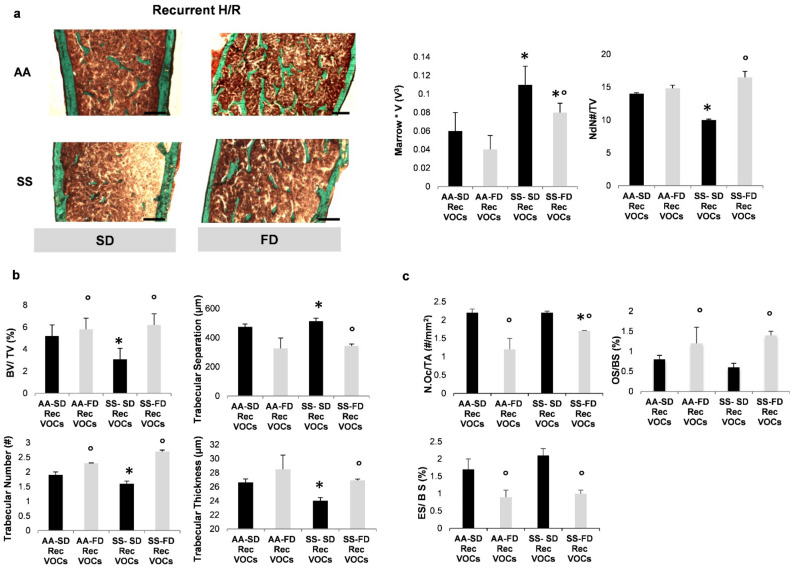
In SCD mice, FD protects bone from recurrent H/R related damage. Quantitative histomorphometric analysis of distal femur of healthy (AA) and sickle cell (SS) mice under either SD or FD supplementation exposed to recurrent H/R. (**a**) **Left panel**. Representative undecalcified section of distal femur stained with Trichoma Goldener’s stain. Microarchitecture was preserved in FD treated mice compared to the SD treated group, expressed as indirect (Marrow Star Volume, M*V) and direct (Node Number, NdN#/TV) parameters (**right panel**). Scale bar: 1 µM. (**b**) Bone Volume (BV/TV) and trabecular parameters were better in the FD treated group compared to the FD group. In particular, the Trabecular Number and Thickness were higher and Trabecular Separation was lower in the FD treated group (**c**) Concerning the cellular activity, the FD group showed reduced osteoclastic number (NOc/TA), increased Osteoblasts Surfaces (OS/BS) and reduced Erosion Surfaces (ES/BS) compared to the SD group. (**a**–**c**) Data are shown as mean ± standard deviation (SD) (*n* = 4; 2 females and 2 males). * *p* < 0.05 compared to AA; ° *p* < 0.05 compared to SD.

As shown in Figure 2a, trabecular structure and bone microarchitecture were preserved in both Rec H/R AA and SS mice fed with FD when compared to Rec H/R SD treated mouse groups, expressed as indirect (Marrow Star Volume, M*V) and direct (Node Number, NdN#/TV) parameters. Bone volume was significantly higher in FD fed SS mice exposed to Rec H/R compared with the SD fed SS mouse group exposed to Rec H/R (Figure 2b).

Trabecular thickness was also higher, whereas trabecular separation was reduced in Rec H/R FD SS mice compared with Rec H/R SD SS animals, demonstrating a protective effect of FD supplementation in SS mice (Figure 2b). Next, we analyzed the balance between bone resorption and bone formation, a key determinant of bone homeostasis. When we evaluated osteoblast/osteoclast function in mice fed with SD or FD diet and exposed to Rec H/R, we found higher numbers of osteoblasts (Obs/BS, Figure 2c) as well as increased osteoid surface (Figure 2c) in Rec H/R FD SS mice. We observed a decreased number of osteoclasts and reduced surface erosion in Rec H/R FD SS mice when compared to Rec H/R SD SS mouse groups. Similar changes, but to a lesser extent, were also observed in Rec H/R FD AA mice compared to Rec H/R SD treated animals (Figure 2b,c).

We also found reduction in bone turnover expressed as AcF compared in Rec H/R FD SS mice compared to Rec H/R SD fed SS animals (Figure S3a), in conjunction with a significant reduction in CTX and of MMP9 activity, both biochemical markers of bone resorption (Figure S3b,c) [6,31].

### 3.3. In SCD Mice Exposed to Recurrent H/R, FD Improves Osteogenesis and Reduces Bone Resorption

**Figure 3:**
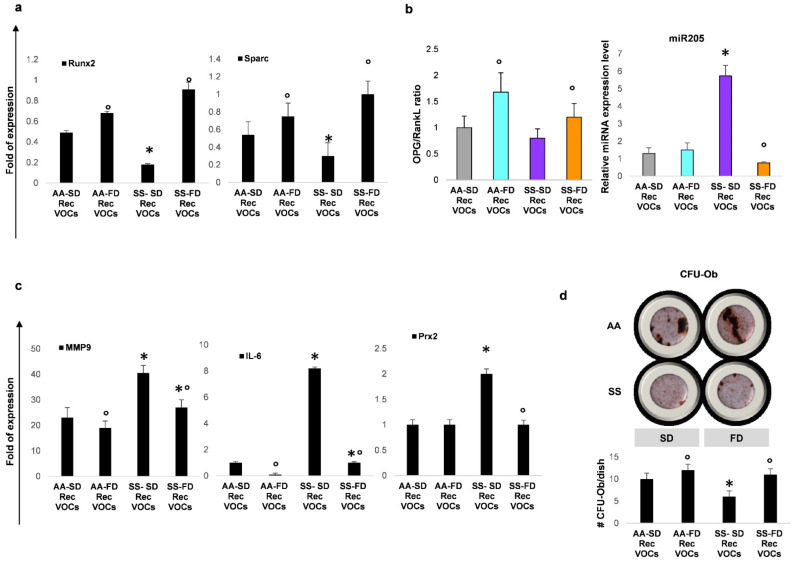
In SCD mice, FD diet beneficially affects osteoblastogenesis and osteogenic commitment. (**a**) Real-time PCR analysis of Runx2 and Sparc gene expression in bone from SD/FD AA and FD SS mice exposed to recurrent H/R (rec H/R). (**b**) **Left panel**. Osteoprogerin (OPG)/RANKL ratio in SD/FD AA and FD SS mice exposed to recurrent H/R (rec H/R). **Right panel**. Bone miR205 expression was downregulated in FD SS mice exposed to rec H/R compared to SD SS animals. (**c**) Real-time PCR analysis of bone interleukin-6 (IL6), matrix metalloproteinase 9 (MMP9) and peroxiredoxin-2 (Prx2) gene expression was reduced in FD SS compared to SD SS mice exposed to recurrent H/R. (**d**) To assess the number of osteoprogenitors, bone marrow derived cells were cultured in vitro under osteogenic differentiation condition. The CFU-Ob obtained from mesenchymal stem cells of AA and SS mouse groups were stained with alizarin red (**upper panel**) and quantified (**lower panel**). By comparing the number of CFU-Ob (colony forming unit-osteoblastic), we observed an increased number of CFU-Ob in FD fed mice, which is in agreement with the increased osteogenic commitment of progenitor cells. (**a**–**c**) Data are shown as mean ± standard deviation (SD) (*n* = 4; 2 females and 2 males). * *p* < 0.05 compared to AA; ° *p* < 0.05 compared to SD. (**d**) Data are shown as mean ± standard deviation (SD); at least six independent experiments have been performed; * *p* < 0.05 compared to AA; ° *p* < 0.05 compared to SD.

The beneficial effect of FD supplementation on bone structure in SS mice exposed to recurrent H/R was also supported by changes in osteogenic markers [6]. We found up-regulation of Runx2 and Sparc gene expression in FD SS mice when compared to SS SD fed animals (Figure 3a).

The OPG/RankL ratio, used to evaluate osteogenesis vs. osteoclastogenesis, was higher in FD SS mice than in the SD SS mouse group (Figure 3b, see also Figure S4). In FD SS mice exposed to recurrent H/R, the improvement of osteogenesis at the expense of osteoclastogenesis was also supported by the downregulation of miR205, which positively modulates osteoclast activity (Figure 3b) [32,33,34]. Since matrix-metalloproteinase (MMP-9) plays an important role in the activation of osteoclasts [31,35,36,37], we evaluated MMP9 expression in bone from FD and SD mouse groups exposed to Rec H/R. As shown in Figure 3c, FD SS mice displayed a significant reduction in MMP-9 expression compared to the SD SS mouse group. This was associated with the down-regulation of both bone IL6 and Prx2 gene expression in FD in FD SS mice exposed to Rec H/R when compared to SD SS mice. Similar changes in Runx2, Sparc gene expression, as well as in the OPG/RankL ratio, and in MMP9 and IL6 gene expression, were also observed in bone from FD AA mice exposed to Rec H/R when compared to the SD AA mouse group (Figure 3a–c). It is worth noting that free fatty acids can activate the G protein coupled receptor (GPR)-40, a receptor for long-chain unsaturated fatty acids, involved in bone remodeling [28,29]. Previous studies have shown that the stimulation of GPR40 prevents bone loss directly acting on OPG and indirectly throughout the modulation of IL-6 mediated local inflammatory response [27,28,29]. Here, we found up-regulation of GPR40 gene expression in bone from both mouse strains treated with FD and exposed to Rec H/R stress, when compared with SD treated animals (Figure S5a).

Taken together, our data indicate that FD prevents Rec-H/R induced bone impairment, modulating systemic and local inflammatory response and oxidation, and promoting an improvement of the osteoblast compartment and a reduction in osteoclast activity. This is in agreement with an increased osteogenic commitment of progenitor cells observed by comparing the number of CFU-Ob (colony forming unit-osteoblastic) in FD and SD from both Rec-H/R healthy and SS mice (Figure 3d).

### 3.4. In SCD Mice Exposed to Recurrent H/R, FD Supplementation Induces Brown Adipogenesis

**Figure 4:**
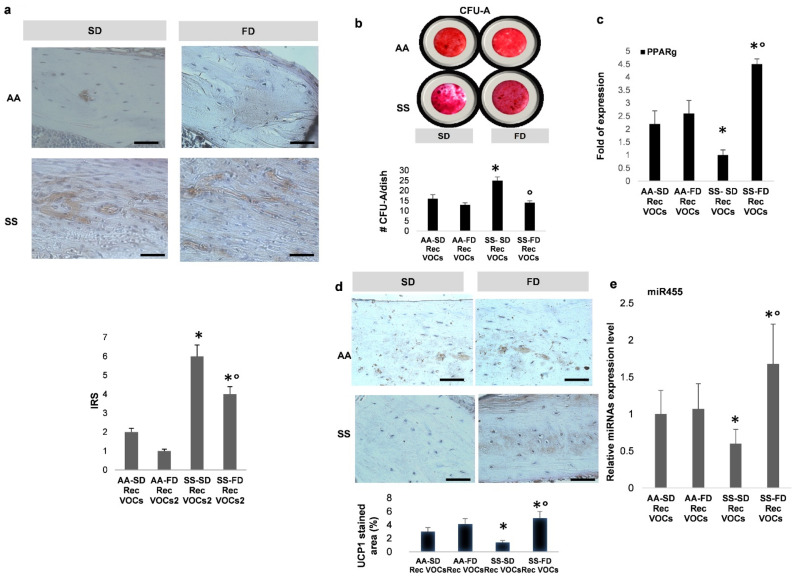
In SCD mice, FD reduces and re-programs adipogenesis from white to brown adipocyte tissue. (**a**) **Upper panel**. Representative images from bone sections of SD/FD AA and SD/FD SS mice exposed to recurrent H/R (rec H/R) stained for Perilipin, a lipid droplet-membrane component. Scale bar: 50 uM. **Lower panel**. IRS expresses the product of Perilipin staining intensity (between negative = 0 and strong = 3) and the percentage of positive Perilipin stained cells. (**b**) To assess the number of adipocytic progenitors, bone marrow derived cells were cultured in vitro under adipogenic (A) differentiation condition. The CFU-A obtained from mesenchymal stem cells of AA and SS mouse groups were stained with Oil Red O (ORO) (**upper panel**) and quantified (**lower panel**). By comparing the number of CFU-A (colony forming unit-adipogenic), we observed a decreased number of CFU-A in FD SS fed mice, which is in agreement with the decreased adipocyte commitment of progenitor cells. (**c**) Real-time PCR analysis of peroxisome proliferator-activated receptor2-γ (PPAR2g) in SD/FD AA/SS mice exposed to rec H/R. (**d**) **Upper panel**. Representative images of bone sections of SD/FD AA and SD/FD SS mice exposed to recurrent H/R (rec H/R) stained for Uncoupling protein-1 (UCP1, brown). Scale bar: 50 uM. **Lower panel**. UCP-1 quantification. (**e**) miR455 expression in bone from SD/FD AA and SD/FD SS mice exposed to recurrent H/R (rec H/R). (**a**,**b**,**d**,**e**) Data are shown as mean ± standard deviation (SD); at least six independent experiments have been performed; * *p* < 0.05 compared to AA; ° *p* < 0.05 compared to SD (**c**,**e**) Data are shown as mean ± standard deviation (SD) (*n* = 4; 2 females and 2 males). * *p* < 0.05 compared to AA; ° *p* < 0.05 compared to SD.

Previous studies in bone remodeling diseases such as osteoporosis have highlighted the key role of altered adipo-osteogenic balance, which contributes to increased marrow fat content at the expense of reduced osteoblastogenesis [38,39,40,41]. Here, we first analyzed the adipogenic compartment in both Rec-H/R AA and SS mice under either SD or FD supplementation. In bone tissue from SS mice, we found increased staining of Perilipin, a lipid droplet-associated protein, when compared to AA animals, independently of diet supplementation (Figure 4a).

However, Perilipin expression was lower in Rec-H/R FD SS mice than in Rec-H/R SD SS animals (Figure 4a). A similar change was also observed in bone from the Rec-H/R FD AA mouse group vs. Rec-H/R SD AA animals (Figure 4a). Molecular analysis of perilipin gene expression in bone from both mouse strains exposed to Rec-H/R stress confirmed the up-regulation of perilipin in SD SS treated mice when compared to FD SS animals (Figure S5b). This agrees with a reduced adipogenic commitment of progenitor cells observed by comparing the number of CFU-A (colony forming unit-adipocytes) in Rec-H/R FD SS mice and Rec-H/R SD SS animals (Figure 3d). It is worth noting that we again observed increased adipogenic commitment of CFU-A in SD Rec-H/R SS mice when compared to FD Rec-H/R SS animals (Figure 4d).

The present data indicate an increased bone marrow adiposity in SS mice compared to healthy animals. This is reduced by FD, which beneficially impacts bone homeostasis and the balance between adipo/osteogenic differentiation of MSCs. To better understand the effects of FD on the adipo/osteogenic differentiation, we evaluated the expression of peroxisome proliferator-activated receptor2-γ (PPAR2γ), a master regulator of adipogenesis, in bone from the different mouse groups [42]. As shown in Figure 4c, we found significant up-regulation of PPAR2γ gene expression in Rec-H/R FD SS mice when compared to either Rec-H/R FD SS mice or Rec-H/R FD/SD AA mice. Since PPARγ has been shown to regulate both white and brown adipocyte differentiation [43,44], we hypothesized that FD might favor browing of white adipocyte tissue [42,45].

**Figure 5:**
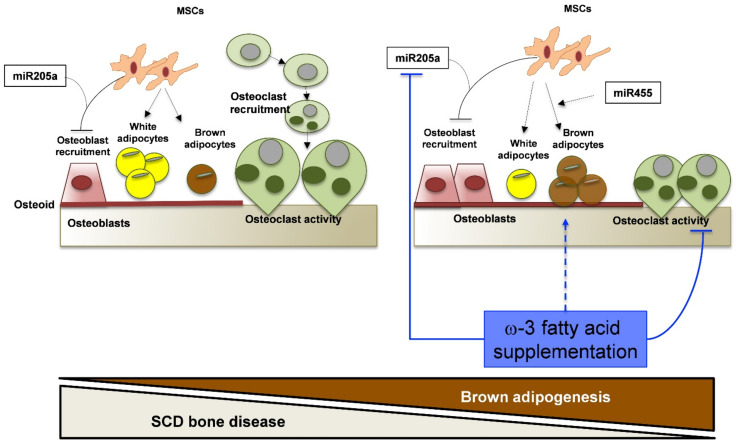
Schematic diagram of the multimodal action of FD supplementation that ameliorates sickle cell bone disease. SCD mice display increased osteoclast recruitment/activity and reduced osteoblastogenesis/activity, ending in reduced osteoid formation and bone loss. miR205a negatively affects osteoblast recruitment in favor of adipogenesis, mainly represented by white adipocyte tissue. FD supplementation reduces osteoclastogenesis/osteoclast activity and downregulates miR205a, favoring osteoblastogenesis/activity. Finally, FD re-programing of adipogenesis resulted in the browning of white adipocyte tissue. The multimodal action of FD protects bone from sickle cell related tissue damage. SCD: sickle cell disease; MSC; mesenchymal stem cells.

Previous studies have shown that uncoupling protein 1 is a key regulator of the metabolic activity of BAT [42,45]. In Rec-H/R FD SS mice, we found increased bone expression of UCP1 when compared to Rec-H/R FD SS animals (Figure 4d). This agrees with the upregulation of UCP1 gene expression in both bone and bone marrow adipocytes from FD SS mice exposed to Rec H/R stress, further supporting the re-programing of adipogenesis in bone compartments of FD SS mice (Figure S6a). Significantly, we also found up-regulation of UCP1 gene expression in visceral fat pads, suggesting a possible systemic effect of omega-3 supplementation on the browning of white adipocyte tissue (Figure S6b). We also investigated the effect of FD on modulation of miR455, a microRNA associated with brown differentiation, acting downstream of BMP7 and inducing the expression of PPARγ [46,47]. In Rec-H/R FD SS animals, the expression of miR455 was increased when compared to either Rec-H/R SD SS animals or AA mouse groups (Figure 4e). These data further support the re-programing of adipogenesis from WAT to BAT in bone compartments and the up-regulation of PPARγ observed in Rec-H/R FD SS mice (Figure 5).

## 4. Discussion

The Western diet is abundant in ω-6 and deficient in ω-3, resulting in an abnormal ω 6/ω 3 ratio, which negatively impacts bone mass density [18,25,48]. Different studies have shown that ω-3 supplementation has a positive effect on bone mineral density and mineral content in human and moue models of bone loss [18,25,48]. In SCD mice, we previously showed that ω-3 diet supplementation (FD) normalizes cell ω 6/ω 3 ratios, reduces systemic inflammation and prevents H/R induced lung and kidney damage.

Here, we show that FD supplementation ameliorates sickle cell bone disease by reducing osteoclastogenesis/activity and increasing osteoblast recruitment/activity. We found down-regulation of Rank and RankL, molecular markers of osteoclast recruitment, as well as of molecules involved in bone resorption such as bone matrix-metalloproteinase-9 (MMP9) or serum CTX. The improvement of bone microenvironment in FD SS mice exposed to Rec-H/R is also supported by the downregulation of the expression of Prx2, a potent antioxidant system. Since bone homeostasis depends on the balance between osteoclastogenesis/osteoblastogenesis, the finding that FD supplementation increased molecular osteogenic markers, such as Runx2 and Sparc, is particularly interesting and is in agreement with the increased number of osteoprogenitor cells, evaluated by analyzing the CFU-Ob. We also demonstrated downregulation of miR205, which negatively affects osteogenic activity by directly targeting Runx2 [32,33,34]. The miR205/Runx2 ratio has been recently proposed as novel marker of bone loss in osteoporosis [33]. We found, in this study, that the miR205/Rux2 ratio is significantly reduced in Rec H/R FD SS mice when compared to Rec H/R SD SS animals (FD SS 0.56 ± 0.08 vs. SD SS 7.2 ± 0.4, *n* = 6; *p* < 0.02), supporting the multimodal action of FD supplementation against SBD. In this context, the FD induced up-regulation of GPR40 is important, possibly playing a pivotal role in the modulation of inflammatory response and in the activation of OPG [28,29]. This again might restrain bone loss, as observed in SS mice under FD treatment.

Previous studies have shown that the balance of osteo-adipogenic differentiation is critical in bone remodeling [23,24,38,39,40,41]. Indeed, increased bone adipose tissue has been described in osteoporosis and other bone loss diseases such as aging and diabetes. This results from a divergent phenotypic commitment of mesenchymal stem cells to adipocytes at the expense of osteoblasts, leading to reduced bone mass [49,50]. In SD SS mice exposed to Rec H/R, we found increased adipogenesis when compared to either Rec H/R FD SS animals or Rec H/R AA mouse groups. This was associated with downregulation of miR205, which decreases adipogenesis and enhances osteogenic activity, but it was apparently in contrast with upregulation of PPARγ expression. PPARγ is the master regulator in adipogenesis [51], but it also involved in the browning process [52], regulating thermogenic capacity in brown adipocytes stimulated by ß-adrenergic factors [53]. We found increased values of UCP1 in Rec H/R FD-mice, confirming that FD supplementation promoted the browning process. Up-regulation of miR455, which has been shown to promote the differentiation of brown adipocytes [54], also supports this conclusion. Our studies in SS mice thus demonstrate that FD supplementation re-programs adipogenesis from WAT to BAT in bone compartments and suggest a possible systemic effect based on the up-regulation of UCP-1 expression in visceral fat pads. Growing evidence supports the beneficial impact of BAT on bone homeostasis either directly or indirectly through the local release of adipokines [46]. Evidence in models of other diseases, such as metabolic syndrome, suggests a possible effect of omega-3 supplementation on mitochondrial biogenesis connected with the browning of white fat tissue [55]. The UCP1 contained in BAT induces energy expenditure by disrupting the mitochondrial respiratory. On the contrary, WAT functions by storing the energy in large lipid droplets [56]. Therefore, the reprograming of bone marrow adipocyte toward a browning fat program affects the lipid accumulation. This last finding has been considered a potential therapeutic tool against different metabolic diseases [56,57]. This additional possible effect of omega-3 supplementation on bone compartments might be explored in future studies using the present model of SBD.

Therefore, our data show that FD supplementation reduces osteoclastogenesis/osteoclast activity and downregulates miR205a, favoring osteoblastogenesis/activity. In addition, FD induces adipogenesis re-programing with the browning of white adipocyte tissue.

## 5. Conclusions

In conclusion, our data indicate that in SCD mice exposed to Rec H/R, an ω-3-enriched diet (i) improves osteoblastogenesis, (ii) decreases osteoclast activity, (iii) modulates the bone inflammatory response, and (iv) re-programs adipogenesis to the browning of white adipose tissue. Our findings provide new insights into the mechanism of action of ω-3 fatty acid supplementation on the pathogenesis of SBD and strengthen the rationale for ω-3 fatty acid dietary supplementation in SCD, as a complementary therapeutic intervention, targeting an amplified inflammatory response and sickle cell-related bone impairment.

## Data Availability

All data reported are showed in this paper or in Supplementary Materials.

## Supplementary Materials

The following are available online at https://www.mdpi.com/article/10.3390/antiox10050799/s1, Figure S1: In SS mice, FD significantly decreased trabecular separation when compared to SD- SS animals. Data are presented as means ± SD (*n* = 4); * *p* < 0.05 compared to AA; ° *p* < 0.05 compared to SD. Figure S2: Hematological parameters in healthy (AA) and sickle cell mice (SS) exposed to recurrent hypoxia/reoxygenation (Rec H/R) supplemented with either SD or FD diet. (**a**) Hct: hematocrit; Hb: hemoglobin; HDW: heterogeneity of red cell distribution. (**b**) peripheral neutrophils. Data are presented as means ± SD, *n* = 6; * *p* < 0.05 compared to AA; ° *p* < 0.05 compared to SD. Figure S3: (**a**) Reduced bone turnover expressed as AcF (Activation Frequency) in control (AA) and sickle (SS) mice exposed to recurrent hypoxia (10 h at 8% O_2_)/reoxygenation and bleeded 11 days after the last hypoxia (Rec VOCs) fed with soy diet (SD) or omega-3 enriched diet (fish oil diet, FD). (**b**) Serum CTX from healthy (AA) and sickle cell mice (SS) exposed to recurrent hypoxia/reoxygenation (Rec H/R) supplemented with SD or FD diet. (**c**) Gelatin zymogram of plasma sample from in control (AA) and sickle (SS) mice exposed to recurrent hypoxia (10 h at 8% O_2_)/reoxygenation and bleeded 11 days after the last hypoxia (Rec VOCs) fed with soy diet (SD) or omega-3 enriched diet (fish oil diet, FD). After 72 hours incubation, transparent bands revealed proteolytic activity of gelatinases. One representative image of 3 with similar results is presented. Colloidal Coomassie stained gel (right panel) was used as loading control. (**a**,**b**) Data are expressed as means ± SD (*n* = 6) * *p* < 0.05 compared to AA; ° *p* < 0.05 compared to SD. Figure S4: Real time PCR analysis of Rank (osteoclastic marker) and RankL (the cytokine inducing osteoclastic activity) gene expression in bone from healthy (AA) and sickle cell mice (SS) exposed to recurrent hypoxia/reoxygenation (Rec H/R) supplemented with FD or SD diet. Data are expressed as means ± SD (*n* = 6) * *p* < 0.05 compared to AA; ° *p* < 0.05 compared to SD. Figure S5: (**a**) Real time PCR analysis of GPR40, free fatty acid receptor 1 (Gpr40), gene expression in bone from healthy (AA) and sickle cell mice (SS) exposed to recurrent hypoxia/reoxygenation (Rec H/R) supplemented with either FD or SD diet Real time PCR analysis of Piripillin2 (PLIN2), a lipid droplet-membrane component gene expression in bone from healthy (AA) and sickle cell mice (SS) exposed to recurrent hypoxia/reoxygenation (Rec H/R) supplemented with either FD or SD diet. (**b**) Real time PCR analysis of GPR40, free fatty acid receptor 1 (Gpr40), gene expression in bone from healthy (AA) and sickle cell mice (SS) exposed to recurrent hypoxia/reoxygenation (Rec H/R) supplemented with either FD or SD diet. Data are expressed as means ± SD (*n* = 6) * *p* < 0.05 compared to AA; ° *p* < 0.05 compared to SD. Figure S6: (**a**) Real time PCR analysis of UCP1 gene expression in bone (left panel) or bone marrow adipocytes (right panel) from healthy (AA) and sickle cell mice (SS) exposed to recurrent hypoxia/reoxygenation (Rec H/R) supplemented with either FD or SD diet. (**b**) Real time PCR analysis of UCP1 gene expression in visceral fat pads from healthy (AA) and sickle cell mice (SS) exposed to recurrent hypoxia/reoxygenation (Rec H/R) supplemented with either FD or SD diet. Data are expressed as means ± SD (*n* = 6) * *p* < 0.05 compared to AA; ° *p* < 0.05 compared to SD.
