# Supplementary material for: Dietary ω-3 Fatty Acid Supplementation Improves Murine Sickle Cell Bone Disease and Reprograms Adipogenesis
Source: Antioxidants (Basel). 2021 May 18;10(5):799. doi: 10.3390/antiox10050799 (PMC8158389; doi:10.3390/antiox10050799)
Supplement: Supplementary file 1 [file antioxidants-10-00799-s001.zip › Supplementary-Figures.pdf]

Figure 1S

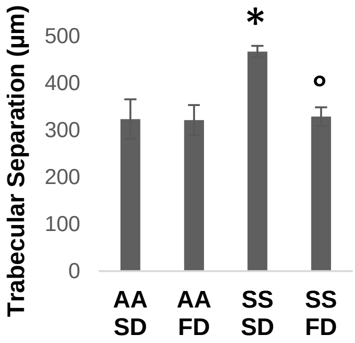

Figure 1S. In SS mice, FD significantly decreased trabecular separation when compared to SD- SS animals. Data are presented as means±SD (n=4); * p<0.05 compared to AA; ° p<0.05 compared to SD

Figure 2S

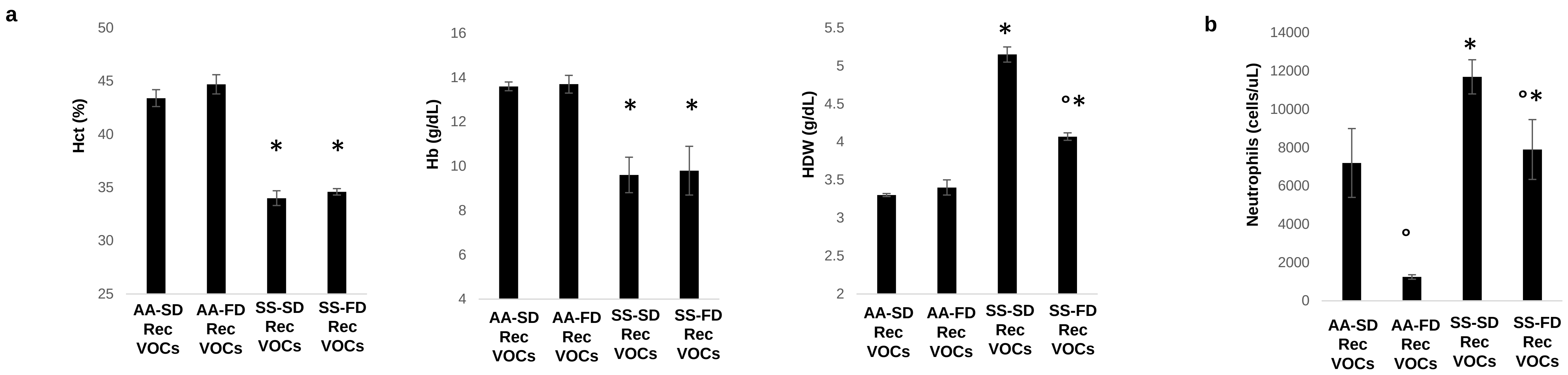

Figure 2S. Hematological parameters in healthy (AA) and sickle cell mice (SS) exposed to recurrent hypoxia/reoxygenation (Rec H/R) supplemented with either SD or FD diet. **(a)** Hct: hematocrit; Hb: hemoglobin; HDW: heterogeneity of red cell distribution. **(b)** peripheral neutrophils. Data are presented as means ±SD, *n*=6; * $p<0.05$ compared to AA; ° $p<0.05$ compared to SD

Figure 3S

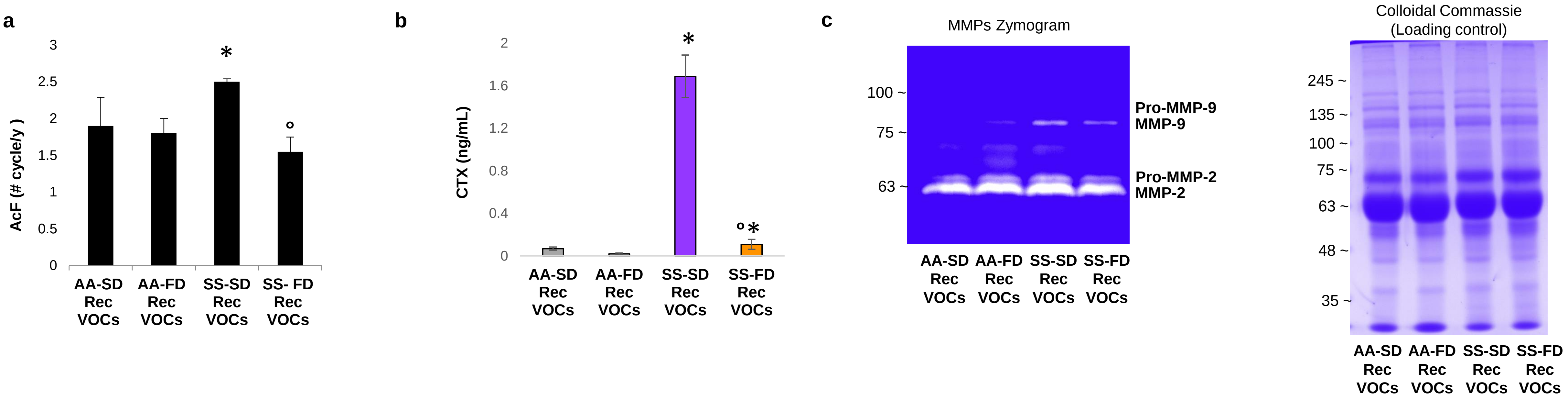

Figure 3S. (a) Reduced bone turnover expressed as AcF (Activation Frequency) in control (AA) and sickle (SS) mice exposed to recurrent hypoxia (10hrs at 8% O2)/reoxygenation and bleeded 11 days after the last hypoxia (Rec VOCs) fed with soy diet (SD) or omega-3 enriched diet (fish oil diet, FD). **(b)** Serum CTX from healthy (AA) and sickle cell mice (SS) exposed to recurrent hypoxia/reoxygenation (Rec H/R) supplemented with SD or FD diet. **(c)** Gelatin zymogram of plasma sample from in control (AA) and sickle (SS) mice exposed to recurrent hypoxia (10hrs at 8% O2)/reoxygenation and bleeded 11 days after the last hypoxia (Rec VOCs) fed with soy diet (SD) or omega-3 enriched diet (fish oil diet, FD). After 72 hours incubation, transparent bands revealed proteolytic activity of gelatinases. One representative image of 3 with similar results is presented. Colloidal Coomassie stained gel (right panel) was used as loading control.

(a-b) Data are expressed as mans ±SD (n=6) * $p<0.05$ compared to AA; ° $p<0.05$ compared to SD

Figure 4S

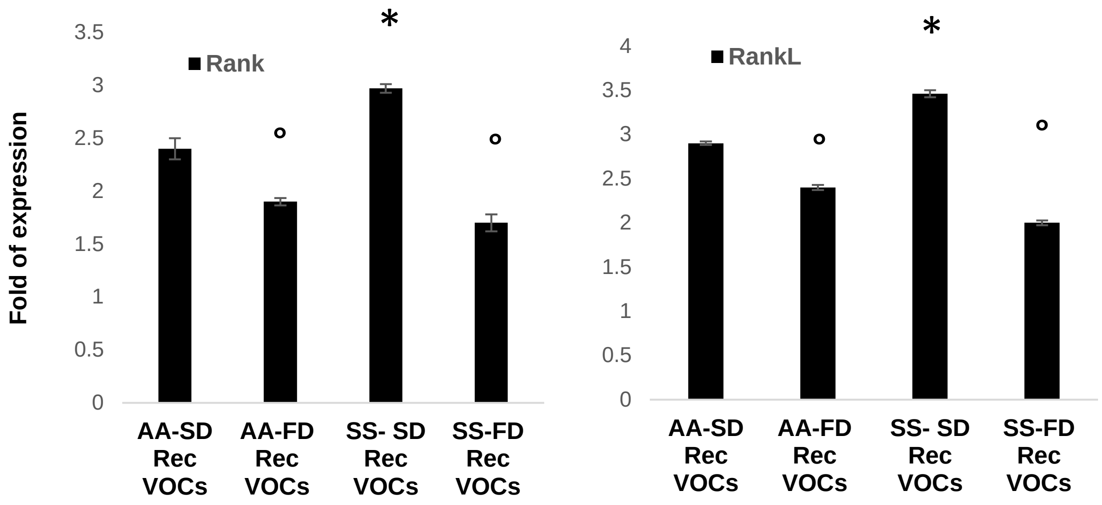

Figure 4S. Real time PCR analysis of Rank (osteoclastic marker) and RankL (the cytokine inducing osteoclastic activity) gene expression in bone from healthy (AA) and sickle cell mice (SS) exposed to recurrent hypoxia/reoxygenation (Rec H/R) supplemented with FD or SD diet.

Data are expressed as mans ±SD (n=6) * $p<0.05$ compared to AA; ° $p<0.05$ compared to SD

Figure 5S

a

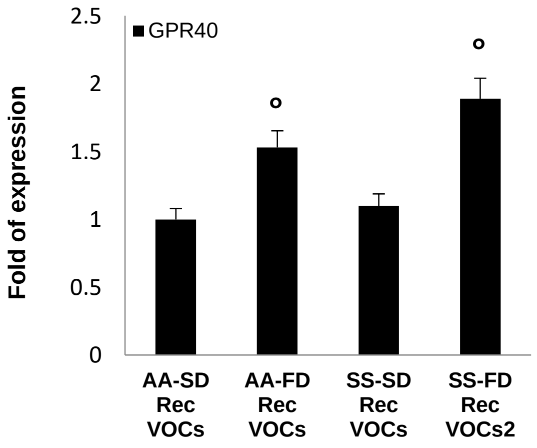

b

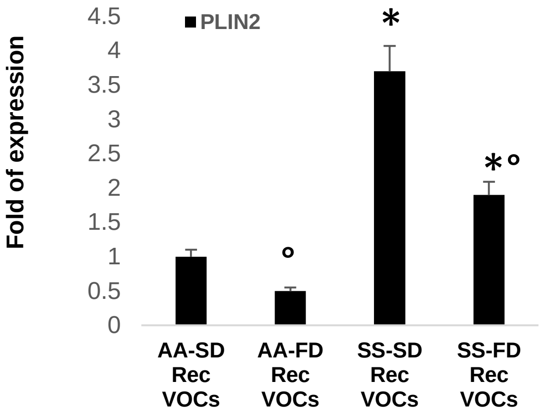

Figure 5S. a. Real time PCR analysis of GPR40, free fatty acid receptor 1 (Gpr40), gene expression in bone from healthy (AA) and sickle cell mice (SS) exposed to recurrent hypoxia/reoxygenation (Rec H/R) supplemented with either FD or SD diet Real time PCR analysis of Piripillin2 (PLIN2) , a lipid droplet-membrane component gene expression in bone from healthy (AA) and sickle cell mice (SS) exposed to recurrent hypoxia/reoxygenation (Rec H/R) supplemented with either FD or SD diet. **b.** Real time PCR analysis of GPR40, free fatty acid receptor 1 (Gpr40), gene expression in bone from healthy (AA) and sickle cell mice (SS) exposed to recurrent hypoxia/reoxygenation (Rec H/R) supplemented with either FD or SD diet

Data are expressed as means ±SD (n=6) * $p<0.05$ compared to AA; ° $p<0.05$ compared to SD

Figure 6S

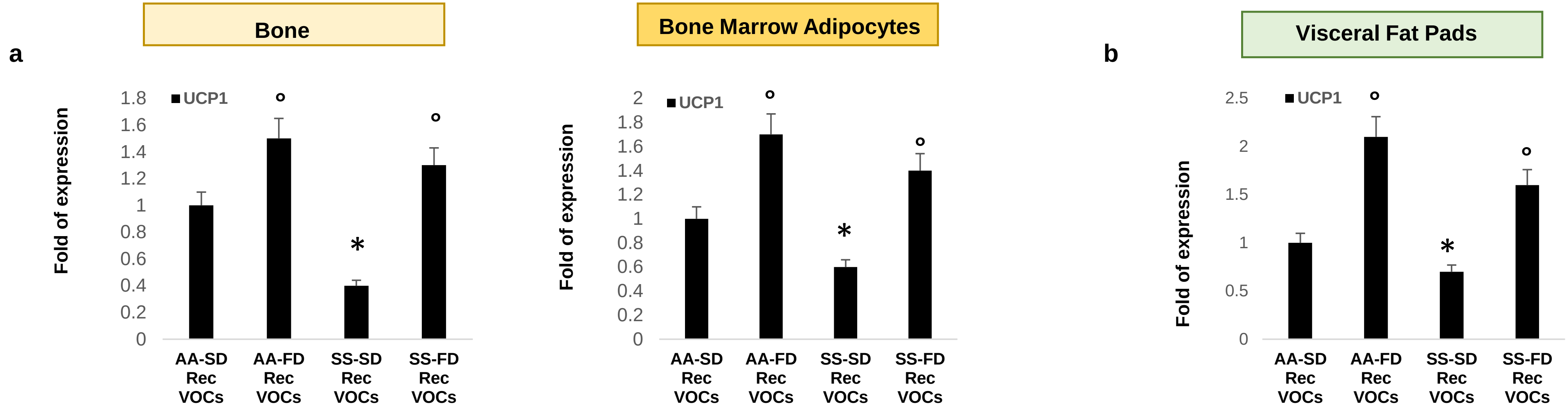

Figure 6S.a. Real time PCR analysis of UCP1 gene expression in bone **(left panel)** or bone marrow adipocytes **(right panel)** from healthy (AA) and sickle cell mice (SS) exposed to recurrent hypoxia/reoxygenation (Rec H/R) supplemented with either FD or SD diet. **b.** Real time PCR analysis of UCP1 gene expression in visceral fat pads from healthy (AA) and sickle cell mice (SS) exposed to recurrent hypoxia/reoxygenation (Rec H/R) supplemented with either FD or SD diet

Data are expressed as means ±SD (n=6) * $p<0.05$ compared to AA; ° $p<0.05$ compared to SD
