# Supplementary material for: Dietary ω-3 Fatty Acid Supplementation Improves Murine Sickle Cell Bone Disease and Reprograms Adipogenesis
Source: Antioxidants (Basel). 2021 May 18;10(5):799. doi: 10.3390/antiox10050799 (PMC8158389; doi:10.3390/antiox10050799)
Supplement: Supplementary file 1 [file antioxidants-10-00799-s001.zip › Supplementary-Method.pdf]

SUPPLEMENTARY METHODS

Mice and design of the study

2 months-old animals were fed for 6 weeks with either the standard AIN-93M purified rodent diet (soy-diet, SD, with n6/n3 ratio of 8:1 - Dyets Inc., Bethlehem, PA), containing 140 g/kg casein, 1.8 g/kg L-cystine, 100 g/kg sucrose, 465.9 g/kg cornstarch, 155 g/kg dextrose, 40 g/kg soybean oil, 0.8 mg/kg t-butylhydroquinone, 50 g/kg cellulose, 35 g/kg mineral mix, 10 g/kg vitamin mix, and 2.5 g/kg choline bitartrate (6)or the ω-3 fatty acid diet (FD), an AIN-93M-based purified rodent diet where all of the calories provided by fat (10%) were replaced by 7.9% from HCO and 2.1% from ω-3 oil (Dyets Inc., Bethlehem, PA). Mice were euthanized and organs immediately removed. Organs were divided into two and either immediately frozen in liquid nitrogen or fixed in 10% formalin and embedded in paraffin for histology. Whenever indicated, mice were exposed to hypoxia (8% oxygen for 10 hours) followed by 3 hours reoxygenation (21% oxygen) (H/R stress) as previously described (6). In order to mimic natural history of sickle cell bone disease H/R stress was carried out 3 times with 3 weeks interval for a total of 3 acute episodes of H/R stress as previously reported (Rec H/R stress).

Measurements of bone homeostasis and turnover.

Bone microarchitecture is the expression of connectivity and it can be evaluated directly through the process of 'skeletonization' of the trabecular bone. This is the measurement of the trabecular profiles and the count of their connections on two dimensional sections by a computed analysis, the so called 'strut analysis'. By this approach, it is possible to obtain a network of two-dimensional components (the struts), which reproduces the trabecular distribution in space, the so called 'Trabecular network'. The link between three or more struts constitutes a node. The number of nodes (NdN) is an index of spatial connectivity. The number of nodes (branch points) and termini (the end points) in a trabecular network, which was skeletonized to facilitate examination of its topologic properties, were analyzed according to (33) In a bone section, the ratio of nodes to termini (Nd/Tm) is an index of spatial connectivity. Thus, higher values correspond to better connectivity (32) In bone histomorphometry, marrow star volume (MSV) is defined as the mean volume of all parts of an object that can be seen unobscured from a random point within the object (34). Higher values of MSV correspond to reduced connectivity (34).

Marrow Star Volume (Marrow *V): is an indirect parameter of microarchitecture and it is defined as the mean volume of all the parts of an object when seen unobscured along uninterrupted straight lines in all directions from random points inside the object. In cancellous bone, MSV provides an estimate of the mean size of the marrow space in three-dimensions and thus reflects connectivity.

To evaluate bone resorption, the number of osteoclast/tissue area (NOc/TA) and erosion surface/bone surface (ES/BS), corresponding to bone covered by eroded cavities, were measured according to international guidelines.

Bone total RNA and microRNA extraction and reverse transcription. Real-time PCR was performed using TaqMan Universal PCR Master Mix (Thermofisher Corporation, Waltham, MA, USA) and TaqMan pre-designed probes for each gene (RUNX2 Mm00501584_m1, RANK Mm00437132_m1, RANK-L Mm00441906_m1, IL6 Mm00446190_m1, PRDX2 Mm04208213_g1, PPARG Mm00440940_m1, UCP (Mm1244861_m1), PLIN2 (Hs04996825-m1 and free

fatty acid receptor 1 (Gpr40): Mm00809442_s1, ACTB Mm02619580_g1, miR203 TM000507, miR455 TM002455, and U6 snRNA MM001973). Gene expression for MMP9 (FW CTTCCAGTACCAAGACAAAG, RV ACCTTGTTCACCTCATTTTG), SPARC (FW CGAGACTTTGAGAAGAACT, RV GGACAGGTACCCATCAATAG) was tested using Power SYBR® Green PCR Master Mix (Thermofisher Scientific, Waltham, MA, USA). Gene expression was normalized to the housekeeping Actin (ACTB) gene, and the relative fold expression differences were calculated. TaqMan SDS analysis software was used to analyze the Ct values. Three independent experiments with three replicates for each sample were performed.

Colony forming units osteogenic and adipogenic assays. The osteogenic medium containing osteogenic stimulatory supplements (10%, StemCell), 10nM dexamethasone, 2mM β-glycerophosphate, 100µM ascorbic acid (StemCell Technologies Inc). The adipogenic differentiation was performed by using 500nM isobutilmetilxantine, 200 µM indomethacin, 1µM dexamethasone and 10 µg/ml insulin in basal medium. For both osteogenic and adipogenic differentiation, the medium was changed every 3 days after initial plating. Pellets from all types of culture were harvested and stored dried a-80°C after three, seven, fourteen and twenty-one days of differentiation. A colony-forming unit–osteoblast (CFU-Ob) or unit-adipocytes assay were conducted as previously reported (6). Briefly, 1×10^6 bone marrow nucleated cells from AA and SS mice in presence of SD or FD were seeded in 60-mm culture dishes in osteogenic or adipogenic differentiation medium (osteogenic: α-minimum essential medium supplemented with 50 µg/mL l-ascorbic acid and 2.0 mM β-glycerophosphate; adipogenic: α-minimum essential medium supplemented with 0.5 mM isobutilmetilxantine, 200 µM indomethacin, 10−6 M dexamethasone and 10 µg/ml insulin) and incubated at 37°C under a humidified atmosphere of 5% CO2. The medium was changed every 2 days, and after 21 days of culture, CFU-Ob colonies were identified by alizarin red staining as previously reported (36). CFU-A colonies were identified by Oil Red O staining as previously reported (36).
